# Case of Transposition of the Thoracic and Abdominal Viscera, with Organic Disease of the Heart

**Published:** 1846-08

**Authors:** Edward R. Squibb


*Case of Transposition of the Thoracic and Abdominal Viscera, with Organic Disease of the Heart.* By Edward R. Squibb, M. D.

For the short history of the following case, and the opportunity of witnessing a post mortem examination of it, the writer is indebted to the kindness of Dr. John S. Rohrer, of Chesnut street, in whose practice it occurred.

The necroscopy was made by this gentleman on the 9th of

April, 1846, twenty one hours after death, in the presence of Professor J. Pancoast, Dr. Squibb, and Dr. Fetters, of Richmond.

The patient, Mr. J. F., æt. 48, was a spare man of medium stature: up to a period, say 3 years, before his death, he had enjoyed tolerable health. After this period he frequently complained of an uneasiness and dull pain in the thorax, which was brought on or augmented by errors in diet, or excitement. A slight bluish tint of the lips and ends of the fingers gave him generally the appearance of coldness or chilliness, and during the latter part of his life, he kept his room heated inordinately. His pulse was hobbling, or intermittent for a year or more, previous to the last few months of his life, when it became much more regular. A marked blowing sound, and a sharp flap were heard over the heart, the locality of which gave rise to difficulty in the diagnosis. He was much troubled by flatulence and irritability of stomach, and by a kind of asthmatic dyspnœa, being obliged to rest in an upright position. This latter was not from any pain or feeling of suffocation which he experienced on lying down, but merely from restlessness and discomfort. A short dry cough also troubled him toward the last, and augmented the laboured feeling about the heart.

About four weeks before death, he was attacked by pericarditis, which yielded without difficulty to the usual treatment.

He was not confined to his bed, or even to his room, immediately preceding his death, but attended to his usual business. A few moments before his death, he was sitting by a very warm stove in a close room, the weather being very mild and pleasant without. He fell suddenly from his chair and was lifted on to a sofa, where he gave a gasp or two and died.

His face and neck were extremely blue, and the veins turgid and prominent, and the pupils dilated to nearly the whole extent.

*Necroscopy.* On removing the sternum, and opening the pericardium, the heart was found to be unusually large, and with its sack exhibiting some marks of recent inflammation. It occupied a position, with relation to the sides of the chest, exactly the reverse of what is usual, the apex being near to the junction of the sixth rib of the right side, with its cartilage, whilst its base was to the left of the median line. The aorta arched towards the right, whilst the arteria innominata and cavæ occupied a position on the left side.

The pulmonic side of the organ was in front and towards the left, the systemic behind and to the right.

The pulmonic side only varied from the natural, in its position and dilatation, except that the eustachian valve was unusually developed, and supported by columnæ carneæ from its free edge. The pulmonary artery was also much dilated, and with the corresponding ventricle and auricle was filled with dark coagulated blood.

The foramen ovale, between the auricles, was patulous to such an extent as to admit the handle of a large scalpel to pass through it with ease. The opening, however, as is usual in such cases, was so oblique as to render the free margins valvular by overlapping.

The systemic auricle was unusually capacious, and its walls much thicker than usual. The corresponding ventricle was also much dilated. The mitral valves were studded throughout by osseous tubercles, and entirely insufficient; and the semilunar valves at the mouth of the aorta in a very similar condition.

The aorta passed down on the right side of the vertebral column, and the ascending cava up on the left. The œsophagus inclining outward, passed through the diaphragm to the right of the median line. The right bronchus was the longer and smaller, and formed a more obtuse angle with the trachea than did the left shorter one. The lung of the left side was divided into three lobes, whilst that of the right had only two.

Ancient adhesions between the pleuræ were numerous, particularly at the posterior portions; and both lungs were somewhat congested. There were also adhesions between the surfaces of the pericardium, particularly over the right ventricle. The larger lobes of the liver with the gall bladder, was found on the left side, as was also the cæcum with its appendix and ascending colon. The spleen much enlarged, was situated to the right; also the pancreas, descending colon and sigmoid flexure. The great end of the stomach lay to the right, and the lesser or pyloric extremity, with the commencement of the small intestine, to the left. The right kidney was of a somewhat clubbed shape, and flattened at the upper larger extremity; it was situated rather higher in the abdominal cavity, than the left one. The left spermatic vein emptied into the ascending cava, sending a branch toward the pelvis of the kidney. The correspoding vein of the right side emptied into the emulgent vein near the pelvis of the right kidney. The origin of the spermatic arteries appeared to correspond with the anomalous termination of the veins, and other minor points, not noted, were probably transposed to an equal extent.

